# Clinical response in advanced non-small cell lung cancer with high PD-L1 expression and *MET* exon 14 skipping mutation: a case analysis of overcoming immunotherapy resistance and literature review

**DOI:** 10.3389/fonc.2026.1807242

**Published:** 2026-05-12

**Authors:** Yefei Ruan, Lin Qiu

**Affiliations:** The Affiliated Yangming Hospital of Ningbo University, Yuyao People’s Hospital, Yuyao, China

**Keywords:** MET14 exon skipping mutation, non-small cell lung cancer, programmed death-ligand 1, savolitinib, tislelizumab

## Abstract

In the precision management of non-small cell lung cancer (NSCLC), identifying driver mutations and evaluating immune-related biomarkers are essential. Yet, clear therapeutic protocols remain absent for tumors that concurrently carry *MET* exon 14 skipping mutations (*MET*ex14) and demonstrate elevated programmed death ligand-1 (PD-L1) expression. This report details the case of a 79-year-old male with a prolonged smoking history who quit 20 years ago, diagnosed with left lung NSCLC. Pathological analysis revealed high PD-L1 expression in the tumor (tumor proportion score 99%), alongside a *MET*ex14 mutation. Initial therapy employed the PD-1 inhibitor Tislelizumab, yielding a progression-free survival (PFS) of 7 months. Treatment was then modified to the *MET* tyrosine kinase inhibitor (*MET*-TKI) Savolitinib, which led to marked tumor reduction. Following discontinuation of Savolitinib due to drug-induced liver injury, Tislelizumab, previously associated with resistance, was reintroduced as a “rechallenge” successfully re-establishing disease control. This distinctive therapeutic sequence provides clinical evidence that “*MET* inhibitors may counteract acquired resistance to PD-1 inhibitors”. Beyond underscoring the value of personalized treatment in the intricate setting of coexisting high PD-L1 expression and *MET*-driven mutations, this case hints at a novel strategy: sequential *MET* inhibitor treatment restore sensitivity to immunotherapy responses by modifying the tumor microenvironment. Coupled with a systematic review of pertinent literature, this article explores the clinical features, therapeutic challenges, potential resistance mechanisms, and management approaches for such patients. It also outlines future research avenues for combination or sequential therapies, aiming to furnish a more holistic reference for clinical decision-making.

## Introduction

Non-small cell lung cancer (NSCLC) is the leading cause of cancer-related death worldwide. Over the last ten years, advances in deciphering tumor development mechanisms have led to notable improvements in therapeutic approaches (1). For advanced NSCLC, current personalized precision medicine relies on three main pillars: targeted treatments for specific driver gene alterations, immunotherapy centered on immune-checkpoint inhibitors(ICIs), and chemotherapy paired with anti-angiogenic agents. Among these, *MET*ex14 is a relatively rare oncogenic driver alteration, found in about 3%–4% of NSCLC cases and more frequently observed in older individuals, women, and non-smokers (2, 3). This genetic change disrupts the ubiquitin-mediated degradation of the *MET* receptor, leading to sustained activation of downstream signaling cascades that foster tumor initiation and progression (4). This alteration is notably enriched in pulmonary sarcomatoid carcinoma, a histologic subtype associated with aggressive behavior and unfavorable prognosis (5). Today, *MET* tyrosine kinase inhibitors designed to target *MET*ex14 (such as capmatinib, tepotinib, and savolitinib) have demonstrated high objective response rates, establishing them as the standard of care for these patients (6, 7).

Meanwhile, for patients whose tumors show a PD-L1 tumor proportion score (TPS) of 50% or higher, monotherapy with a PD-1/PD-L1 monoclonal antibody has become the established first-line treatment (8, 9). However, when both high PD-L1 expression and *MET*ex14 mutation, two molecular characteristics with well-defined therapeutic relevance, coexist in the same individual, a distinct clinical conundrum arises: should targeted therapy or immunotherapy take precedence? At present, no clear consensus or guideline exists to direct management strategies for such “double-positive” cases. Some available data indicate that patients harboring *MET* mutations generally respond poorly to immunotherapy and experience shorter progression-free survival (10). Nevertheless, recent investigations have highlighted that in a subgroup with exceptionally high PD-L1 expression (TPS≥80%), immunotherapy might show efficacy comparable to or even surpassing that of targeted therapy, thereby questioning the conventional wisdom of prioritizing targeted treatment (11, 12). Hence, determining the optimal therapeutic approach for this rare yet clinically important patient group has emerged as a pressing necessity.

Herein, we present a challenging case of advanced NSCLC harboring both a high PD-L1 TPS and a *MET*ex14 mutation, illustrating a unique sequential treatment strategy and raising important questions regarding therapeutic interplay and resistance mechanisms.

## Case report

### Patient information

The patient is a 79-year-old male with a significant smoking history, who quit smoking twenty years ago. In November 2022, a chest CT scan identified a space-occupying lesion in the right upper lung lobe, prompting referral to our institution for further evaluation. Upon admission, physical examination revealed a barrel-shaped chest, diminished breath sounds bilaterally, and no other notable positive findings.

### Clinical examination and diagnosis

**Figure 1:**
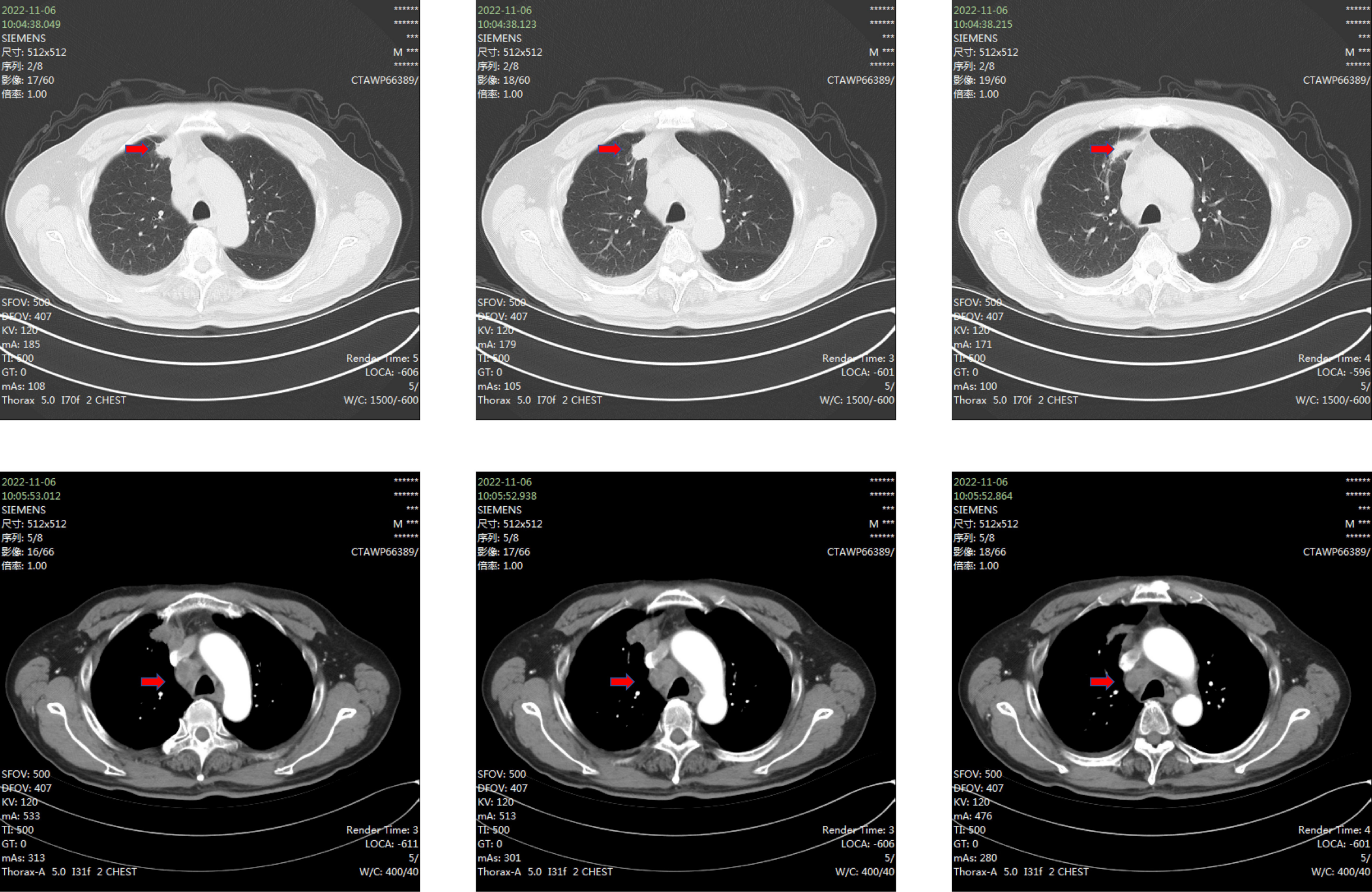
Red arrows indicate the primary lung lesion and metastatic lymph nodes.

**Figure 2:**
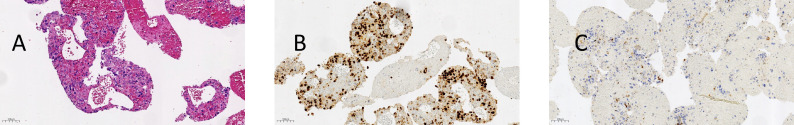
**(A)** hematoxylin and eosin stain 100x. **(B)** IHC for TTF-1 100x: +. **(C)** IHC for P40 100x: individual+.

Contrast-enhanced chest CT demonstrated a solid nodule measuring approximately 20×27 mm in the right upper lobe, accompanied by mediastinal lymphadenopathy (**Figure 1**). An ultrasound-guided transbronchial needle aspiration biopsy of the 4R mediastinal lymph nodes was performed via bronchoscopy to establish a pathological diagnosis. Histopathological examination confirmed non-small cell lung carcinoma. Subsequent immunohistochemical analysis showed tumor cells with diffuse strong positivity for thyroid transcription factor-1 (TTF-1), while P40 staining was weakly positive in only a minority of cells, supporting a diagnosis of lung adenocarcinoma (**Figure 2**). For tumor staging, whole-body bone scintigraphy (ECT) was conducted, revealing evidence of bone metastasis. PD-L1 immunohistochemical testing of the tumor tissue indicated high expression, with a tumor proportion score (TPS) of 99%. Additionally, molecular pathological analysis of the tumor tissue via next-generation sequencing identified the following driver gene mutations: *MET* exon 14 skipping mutation, *TP53* p.R273H point mutation, and *MET* exon 16 p.H1094Y point mutation.

### Treatment process

**Figure 3:**
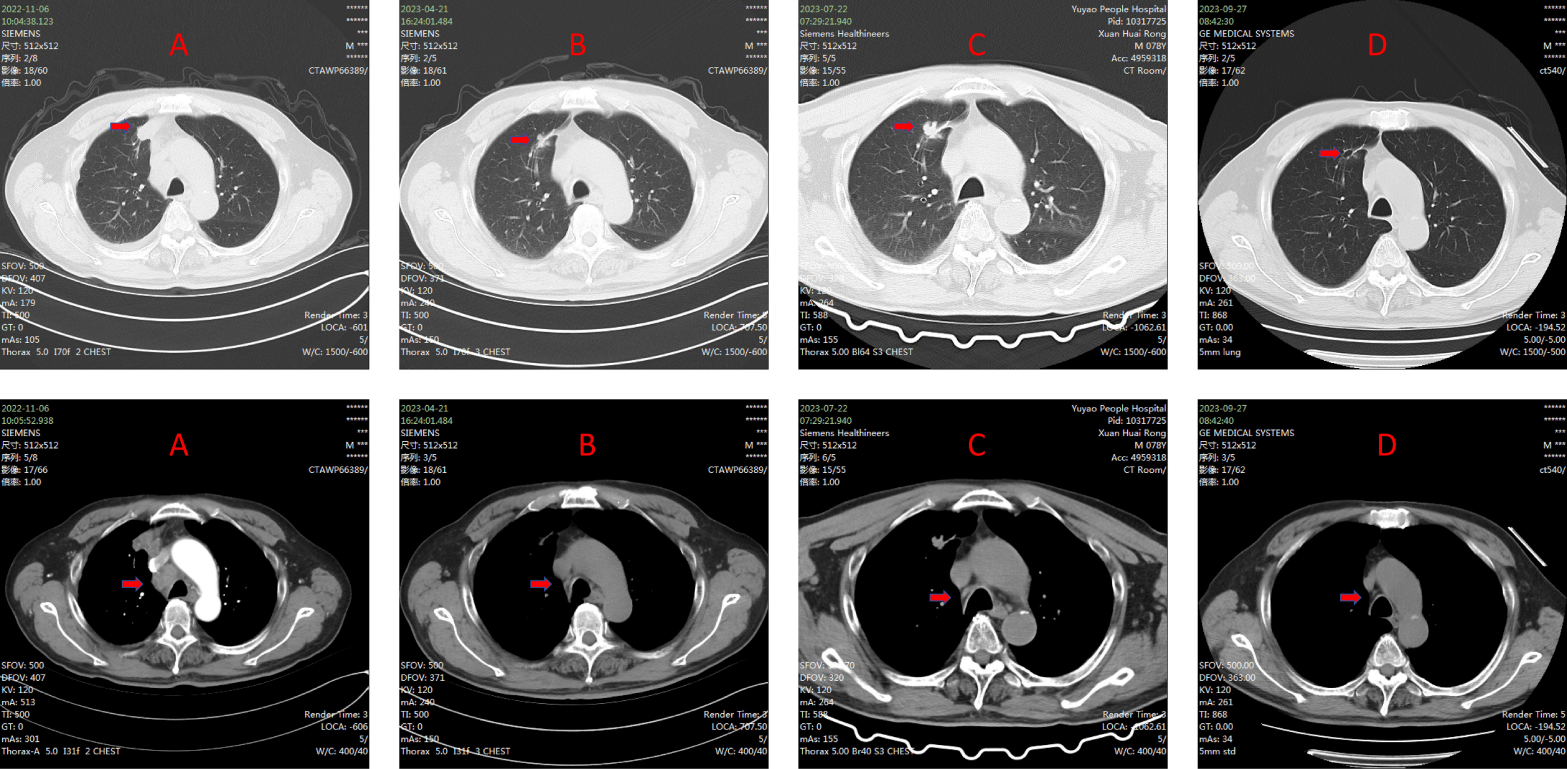
**(A)** The computed tomography (CT) scan before treatment. **(B)** the CT scan after 6 cycles of administration of tislelizumab, showing that the tumor significant reduction. **(C)** The CT scan after 8 cycles of administration of tislelizumab, showing that the tumor had increased in size. It indicates that the tumor has developed resistance to tislelizumab. **(D)** the CT scan conducted two months after the administration of savolitinib showed that the tumor shrank again.

Beginning in December 2022, the patient was treated with Tislelizumab monotherapy, administered every three weeks at a dose of 200 mg. After six cycles of treatment, imaging follow-up demonstrated a reduction in lesions, with efficacy assessed as partial response (**Figure 3A** versus **B**). The patient then continued this regimen as maintenance therapy, completing a total of eight cycles. However, subsequent imaging follow-up revealed locoregional disease progression, indicated by an increase in the size of the primary lung lesion (**Figure 3C**).

Given the detection of a *MET*ex14 through driver gene testing, the clinical treatment approach was modified to targeted therapy, initiating oral Savolitinib at a daily dose of 400 mg. Two months after treatment initiation, follow-up chest CT showed a marked reduction in lesions, indicating clear therapeutic benefit (**Figure 3D**). Nevertheless, during Savolitinib treatment, the patient developed severe drug-induced liver injury, meeting criteria for Grade 3 (severe) hepatotoxicity according to CTCAE v5.0. Consequently, Savolitinib was promptly discontinued, and liver-protective and enzyme-lowering treatments were commenced. After over a month of symptomatic supportive care, the patient’s liver function parameters returned to the normal range.

Following further discussion with the patient and obtaining informed consent, anti-tumor therapy with Tislelizumab was resumed on November 29, 2023.

### Follow-up and clinical outcomes

**Figure 4:**
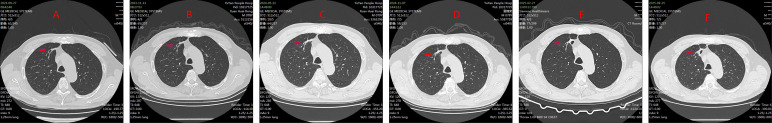
**(A)** The CT scan after administration of savolitinib. **(B)** the CT scan rechecked before using tislelizumab again. **(C–F)** during the second treatment with tislelizumab, the CT scan shows that the tumor remains stable.

**Figure 5:**
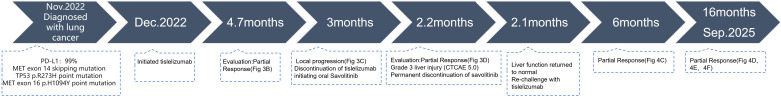
Treatment timeline.

After resuming Tislelizumab therapy, routine imaging surveillance revealed stable lesions with no evidence of disease progression (**Figure 4**). Currently, the patient remains in stable condition and continues to receive treatment under close monitoring. The most recent disease reassessment on September 25, 2025 showed sustained disease control, resulting in a total follow-up duration of thirty-three months from initial treatment. The detailed treatment timeline is shown in **Figure 5**.

### Literature review and discussion

The patient described in this report is a 79-year-old male with a long-standing history of tobacco use. This feature stands in notable contrast to the typical profile outlined in prior studies, where *MET*ex14 are more commonly observed in older, female, and non-smoking individuals. Although evidence suggests that the efficacy of ICIs may be reduced in *MET*ex14-mutated NSCLC (10, 13), this patient achieved a PFS of 7 months with first-line tislelizumab. This outcome may be related to the high TPS of 99% for PD-L1 expression on tumor cells. These findings indicate that in cases of extremely high PD-L1 expression, ICIs may remain a viable initial treatment option. Furthermore, this suggests that the tumor’s response to treatment may be influenced by multiple factors, including but not limited to PD-L1 expression level (14), Tumor Mutational Burden (TMB) (15), and co-occurring genetic alterations (16) (such as the *TP53* mutation found in this case). Recent investigations have similarly noted that within the subgroup of patients exhibiting very high PD-L1 expression (TPS≥ 80%), ICIs have demonstrated considerable therapeutic promise (11, 12).

The distinctiveness of this case is further evident in its subsequent management. After acquired resistance to tislelizumab emerged, switching to the *MET*-TKI savolitinib led to marked tumor reduction, indicating that high PD-L1 expression does not compromise the efficacy of targeted agents. More importantly, upon discontinuation of savolitinib due to drug-induced liver injury, re-administration of tislelizumab continued to maintain disease stability. This unique treatment sequence raises a critical clinical question: why did immunotherapy regain efficacy following discontinuation of targeted therapy? Existing literature has more frequently documented primary resistance to ICIs in patients with *MET*ex14 mutations or the potential heightened risk of ICI-related adverse events following *MET*-TKI therapy (17), whereas case reports successfully overcoming immune resistance through sequential treatment remain relatively scarce. Moreover, some case reports suggest that the combined use of anti-CTLA-4 and anti-PD-1 antibodies might overcome PD-1 inhibitor resistance in *MET*ex14-mutated NSCLC (18). A plausible hypothesis explaining the success of the immunotherapy re-challenge in this case is that savolitinib, by effectively inhibiting the *MET*ex14-driven oncogenic pathway, may have modulated the tumor microenvironment (TME). Preclinical and clinical evidence suggests that *MET* signaling may promote an immunosuppressive TME through mechanisms such as recruiting myeloid-derived suppressor cells or upregulating alternative immune checkpoints (19). Therefore, *MET*-TKI treatment may have partially “reset” the TME, making it more conducive to T-cell-mediated cytotoxicity, thereby restoring sensitivity to PD-1 blockade. However, this mechanistic explanation remains speculative, and research data are limited.

The complexity of this case necessitates consideration of other contributing factors. First, the tumor’s molecular profile is complex, with concurrent TP53 and *MET*ex14 mutations. In the absence of variant allele frequency data, assessing clonal dynamics and determining whether the *MET*ex14 alteration is a truncal driver event is challenging. Second, the patient’s significant smoking history contrasts with the typical *MET*ex14 mutated population and may have contributed to a unique tumor mutational burden and immunogenic landscape, potentially confounding the treatment response pattern. Third, the clinical context of the tislelizumab re-challenge is unique: savolitinib was discontinued due to toxicity while the tumor was in a state of response, not radiographic progression. This minimal residual disease (MRD) scenario may represent a biological background where immune surveillance is more effective, differing from the standard scenario of TKI treatment progression. This exemplifies the need for careful clinical judgment in individualized oncology.

Through the unique and effective treatment sequence of “targeted therapy after immunotherapy resistance, followed by re-challenge with immunotherapy,” this case provides valuable insights for individualized treatment in NSCLC patients with high PD-L1 expression and concurrent *MET*ex14 mutation. Its core significance lies in providing preliminary clinical evidence that *MET*-TKIs may reverse acquired resistance to PD-1 inhibitors, offering a new perspective on treatment strategies for this specific patient subgroup.

The management of this case underscores the intricacies involved in clinical decision-making and the potential quandaries encountered in NSCLC patients who exhibit both high PD-L1 expression and *MET*ex14 mutations. While existing national and international guidelines typically advocate targeted therapy as the frontline standard for individuals with *MET*ex14 mutations (3, 6, 20–22), in real-world clinical practice, the selection of initial treatments for patients continues to diverge significantly. This variability stems from a multitude of factors, including drug availability, disparities in regional approvals, and clinician preferences. Furthermore, this case and recent research data indicate that for patients with extremely high PD-L1 expression (TPS ≥ 80%), the median overall survival (mOS) with immunotherapy (with or without chemotherapy) is significantly better than that with single-agent *MET*-TKI treatment, suggesting that immunotherapy can also be considered an effective initial treatment option (11, 12). This finding challenges the singular model of “targeted therapy first” and underscores the importance of individualized treatment decisions based on multidimensional biomarkers. A key clinical misconception is that focusing solely on driver gene mutations while overlooking extremely high PD-L1 expression may lead patients to miss the opportunity to benefit from immunotherapy. Additionally, it must be anticipated that resistance to *MET*-TKIs will eventually develop (23, 24). Therefore, for such “double-positive” patients, comprehensive molecular pathology and immune profiling analysis forms the foundation for developing precise treatment strategies.

The *MET*-TKI mediated immune re-sensitization mechanism proposed through our observation in this case is hypothetical, and the interpretation is inherently limited by the nature of a single retrospective case report. Validation through prospective studies and translational research is needed. Additionally, this study lacks baseline TMB data to support the potential reasons for the initial effectiveness of immunotherapy, and no re-biopsy was performed at disease progression to analyze specific resistance mechanisms. Therefore, the generalizability of its conclusions is limited, and whether this experience applies to a broader *MET*ex14 mutated NSCLC population remains uncertain.

## Conclusion

This case report, along with a literature review, indicates that for patients with advanced non-small cell lung cancer exhibiting exceptionally high PD-L1 expression levels and concurrent *MET*ex14 skipping mutations. Therefore, clinical treatment decisions should be highly individualized. Immunotherapy remains a viable option as part of first-line treatment. The observed phenomenon of “successful reversal of acquired resistance to PD-1 inhibitors through *MET* tyrosine kinase inhibitors” offers a novel clinical perspective and practical foundation for overcoming immunotherapy resistance.

In the future, by further deepening basic research and clinical translational exploration, we aim to identify biomarkers that can predict the response to immunotherapy or targeted therapy, thereby clarifying the target population for prioritizing immunotherapy or targeted therapy among “double-positive” patients; utilize multi-omics technologies (such as single-cell RNA sequencing and spatial transcriptomics) to deeply elucidate the molecular and immunological mechanisms by which MET inhibitors reverse immunotherapy resistance; explore new combination or optimized sequential treatment modalities of MET-TKIs and ICIs, while properly managing potential overlapping toxicities (such as hepatotoxicity and interstitial pneumonia). This will contribute to developing more optimized and precise treatment strategies for this specific patient population.
